# A molecular signature predicts hematologic evolution in polycythemia vera patients

**DOI:** 10.1038/s41375-025-02660-0

**Published:** 2025-06-18

**Authors:** Olivier Mansier, Eric Lippert, Lina Benajiba, Dana Ranta, François Girodon, Jean-Christophe Ianotto, Aurélie Chauveau, Lydia Roy, Françoise Boyer, Clémence Médiavilla, Suzanne Tavitian, Marion Divoux, Mélinda Fanet, Ivan Sloma, Véronique De Mas, Guillaume Denis, Christopher Nunes Gomes, Claire Calmettes, Fiorenza Barraco, Sarah Huet, Fabienne Vacheret, Mélanie Mercier, Anne Parry, Laurence Legros, Juliette Soret-Dulphy, Joris Argentin, Léa Sureau, Emmanuelle Verger, Corentin Orvain, Jérémie Riou, Jean-Jacques Kiladjian, Bruno Cassinat, Valérie Ugo, Damien Luque Paz, on behalf of the French Intergroup of Myeloproliferative Neoplasms (FIM), Olivier Mansier, Eric Lippert, Lina Benajiba, Dana Ranta, François Girodon, Jean-Christophe Ianotto, Aurélie Chauveau, Lydia Roy, Françoise Boyer, Clémence Médiavilla, Suzanne Tavitian, Ivan Sloma, Guillaume Denis, Claire Calmettes, Fiorenza Barraco, Sarah Huet, Fabienne Vacheret, Mélanie Mercier, Anne Parry, Laurence Legros, Juliette Soret-Dulphy, Léa Sureau, Emmanuelle Verger, Corentin Orvain, Jean-Jacques Kiladjian, Bruno Cassinat, Valérie Ugo, Damien Luque Paz, Véronique De Mas

**Affiliations:** 1 CHU Bordeaux, Laboratoire d’Hématologie, Bordeaux, France; 2 Université de Bordeaux, Inserm, U1034 Bordeaux, France; 3 CHU Brest, Service d’Hématologie Biologique, Brest, France; 4 Univ Brest, Inserm, EFS, UMR 1078, GGB, Brest, France; 5 INSERM U944/CNRS UMR7212, Université de Paris, Hôpital Saint Louis APHP, Paris, France; 6 CHU Nancy, Hématologie clinique, Nancy, France; 7 CHU Dijon, Laboratoire d’Hématologie, Dijon, France; 8 CHU Brest, Hématologie et Hémostase Clinique, Brest, France; 9 CH Cornouaille, Laboratoire d’Hématologie, Quimper, France; 10 AP-HP, Hôpital Henri Mondor, Service d’Hématologie, Créteil, France; 11 Université Paris Est Créteil (UPEC), Faculté de Santé, F-94000 Créteil, France; 12 CHU Angers, Service des Maladies du Sang, Angers, Fédération Hospitalo-Universitaire Grand-Ouest Acute Leukemia, FHU-GOAL et Université d’Angers, Inserm UMR 1307, CNRS UMR 6075, Nantes Université, CRCI2NA Angers, France; 13 CHU Bordeaux, Hématologie clinique, Bordeaux, France; 14 CHU Toulouse, Service d’Hématologie, Institut Universitaire du Cancer de Toulouse Oncopole, Toulouse, France; 15 CHU Nancy, Laboratoire d’Hématologie, Nancy, France; 16 AP-HP, Hôpital Henri Mondor, Département d’Hématologie et Immunologie, F-94010 Créteil, France; 17 Université Paris Est Créteil, INSERM, IMRB, F-94010 Créteil, France; 18 Université de Toulouse III, CHU Toulouse, Laboratoire d’Hématologie, Institut Universitaire du Cancer de Toulouse Oncopole, Toulouse, France; 19 CH Rochefort, Service d’Hématologie, Rochefort, France; 20 CH Cholet, Hématologie Clinique, Cholet, France; 21 CH Périgueux, Hématologie Clinique, Périgueux, France; 22 Hospices Civils de Lyon, Hôpital Lyon Sud, Hématologie clinique, Pierre-Bénite, France; 23 Hospices Civils de Lyon, Hôpital Lyon Sud, Laboratoire d’Hématologie, Pierre-Bénite, France; 24 CH Perpignan, Service d’Hématologie, Perpignan, France; 25 CH Bretagne Atlantique, Service d’Hématologie, Vannes, France; 26 CH Annecy, Hématologie, Annecy, France; 27 APHP Bicêtre, Hématologie clinique, Le Kremlin Bicêtre, France; 28 CHU Angers, Laboratoire d’Hématologie, Angers, France; 29 Université Paris Cité, APHP, Hôpital Saint-Louis, Laboratoire de Biologie Cellulaire, Paris, France; 30 Univ Angers, CHU Angers, Inserm, CNRS, MINT, SFR ICAT, Angers, France; 31 Univ Angers, Nantes Université, CHU Angers, Inserm, CNRS, CRCI2NA, F-49000 Angers, France

**Keywords:** Myeloproliferative disease, Cancer genomics

## Abstract

Genetic analyses have been included in scoring systems to improve the prognostic stratification of hematologic malignancies. Until now, molecular risk scores have not been included into the practical management of patients with polycythemia vera (PV). In this work, we studied 439 PV patients recruited from 15 French centers and described their mutational landscape using high-throughput sequencing. We detected an additional mutation in 53.3% of patients, 22.7% of them having 2 or more mutations. A Bayesian approach identified preferential associations between mutations. Based on these associations, we identified high molecular risk abnormalities in PV (PV-HMR), consisting in mutations in *SRSF2*, *IDH1/2*, *EZH2* or *NFE2* genes, copy number variations (CNV) and carrying 2 or more non-driver mutations. These PV-HMR were associated with decreased overall survival (OS) and/or transformation-free survival (TFS). Notably, *ASXL1* mutations were not associated with a pejorative impact on OS or TFS when isolated. Based on these results, we developed a genomic 3-tier classification that efficiently predicted OS and more importantly TFS independently of age, sex, history of thrombosis and leukocyte and platelet counts. This model outperformed the IWG-PV and MIPSS-PV scoring systems in predicting the hematologic evolution of PV patients, which was confirmed in 2 external cohorts.

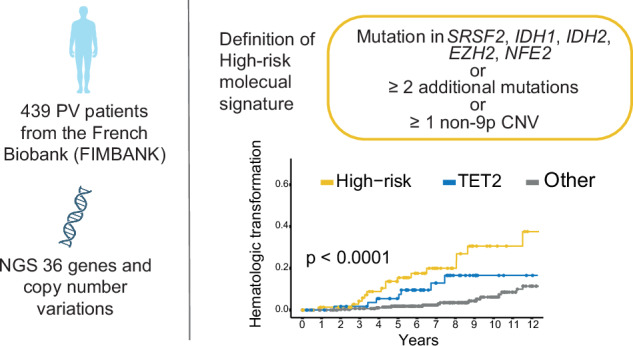

## Introduction

Philadelphia-negative myeloproliferative neoplasms (MPN) are hematologic malignancies characterized by an excessive production of mature blood cells. They are the consequence of the acquisition of somatic mutations, the most frequent being *JAK2*V617F. MPN include different entities, mainly polycythemia vera (PV), essential thrombocythemia (ET) and primary myelofibrosis (PMF) [1, 2].

Although sometimes considered as indolent diseases, PV and ET are associated with a reduced survival compared to the general population [3] because of 2 types of complications: thrombo-embolic events, which are the most frequent, and progression to secondary myelofibrosis (SMF) and/or secondary acute leukemia (sAML), which generally occur at longer term. In a long-term follow-up cohort of patients with PV, the main cause of death in patients younger than 65 years at the time of diagnosis was hematologic transformation (54%), while vascular events accounted for 15% of deaths [4].

Adequate management of PV patients requires risk prediction for these 2 types of complications However, available prognostic stratification is imperfect. Age, history of thrombosis have been associated with both thrombosis and decreased survival [4], while leukocytosis, detection of *JAK2*V617F or low red blood cell distribution width [5] have been associated with an increased risk of thrombosis. Recently, some clinical parameters such as general symptoms (fatigue, pruritus, sweating, bone pain, weight loss, and fever [6]), comorbidities or body mass index [7] have also been shown to impact PV prognosis. Finally, some biological factors such as leukocytosis, neutrophil/lymphocyte ratio or *JAK2*V617F allele burden have been associated with decreased survival and/or increased risk of hematologic transformation [8, 9]. However, there is no effective prognostic scoring system to predict the risk of progression.

Hematologic transformations of MPN are associated with the detection of additional genetic alterations. Generally, the clone carrying these additional mutations is already detectable at diagnosis, but expands at the time of MPN transformation [10, 11]. The detection of additional mutations during chronic phase has improved the risk stratification in PMF patients, with the definition of “high molecular risk” (HMR) mutations and the development of several scoring systems to guide treatment management [12–16]. This is not the case for PV, with only two studies describing the molecular landscape of PV patients. The first evaluated a cohort of 133 patients and showed an adverse prognosis for *ASXL1*, *SRSF2* and *IDH2* mutations. These results were overall confirmed on an external validation cohort of 215 patients [17]. The second study was performed in 146 patients and identified an association between *SRSF2* mutations and decreased overall survival leading to its inclusion in the MIPSS-PV scoring system [18]. However, no association between additional mutations and the risk of transformation to SMF or sAML was observed in this study [18]. Thus, it is not clear whether molecular analysis could provide relevant information to refine risk stratification of PV patients and predict hematologic transformation.

In the present study, we sought to demonstrate that molecular analysis improves our ability to predict hematologic outcomes and overall survival in PV patients. By studying 439 PV patients, we identified 3 groups of patients based on a simple, targeted genetic analysis. These genetic data were integrated together with clinical and biological data into a multistate model that outperformed existing scoring systems to predict hematologic transformation. Our data demonstrate that molecular analysis provides additional information to refine prognostic stratification of PV patients.

## Patients and methods

Design and method of the study were performed according the REporting recommendations for tumour MARKer prognostic studies (REMARK) (see Supplementary Data).

### Patients and samples

Patients were recruited from the French Intergroup of Myeloproliferative Neoplasms (FIM) national database (BCB FIMBANK) if they had a diagnosis of PV between 2005 and 2018 according to the World Health Organization 2008 or 2016 classifications. Peripheral blood DNA from 471 unselected patients from 15 French hospitals were centralized at Angers hospital for high-throughput molecular analysis. Overall, 439 patients were included in the analysis after exclusion of 32 patients (see details in Supplementary Data). All samples were collected at diagnosis or during the first year after initial diagnosis and consisted of DNA derived from whole blood (84.5%) or purified blood granulocytes (15.5%). Clinical and biological information at diagnosis and during follow-up was extracted from the BCB FIMBANK database. Patients provided their informed consent to be included in the BCB FIMBANK and the present project has been registered by the French Data Protection Authority (Commission Nationale de l’Informatique et des Libertés, ar22-0062v0).

### NGS sequencing and analysis

For targeted high-throughput sequencing, we used a custom RNA-baits panel designed to cover all exons of 36 genes involved in myeloid malignancies or previously described as mutated in MPN. The targeted genes are listed in the **Supplementary Data**. Bioinformatic tools were used to call and annotate variants and to detect Copy Number Variations (CNV) on chromosomes 1q, 5, 7, 8, 9p, 13q, 17p and 20q. The bioinformatic pipeline is detailed in the **Supplementary Data**. Only exonic or splicing mutations (donor and acceptor sites) with a variant allele frequency ≥2% and not described as common polymorphisms (*ie* ≥ 1% in general population) were considered. As previously used by our group [16], variants were classified according to their putative pathogenic effect as pathogenic, likely pathogenic, or variant of unknown significance according to standard guidelines (details in **Supplementary Data**). Of note, mutations of unknown significance with a minor allele frequency ≥0.01% in the general population were considered as rare polymorphisms and removed. All samples were interpreted independently by 2 trained molecular biologists. All discordant results were then collectively reviewed and discussed. Only pathogenic and likely pathogenic mutations were kept for statistical analysis of prognosis.

### Validation cohorts

In order to validate our results, we used two independent external cohorts of patients: one already published by Grinfeld et al. [19] with 316 PV patients, and the second from St-Louis Hospital including 365 PV patients (IRB00006477, CER-2020-55).

### Statistics

A Bayesian network analysis combined with hierarchical clustering analysis (HCA) was performed to characterize homogeneous clusters of genes. The association between mutational status and overall survival and hematologic transformations was studied using a multi-state model. Correction of *p*-values was performed with the Benjamini-Hochberg procedure. The detailed methodology is described in the Supplementary Data.

## Results

### Description of the cohort

We included 439 cases of PV patients in the analysis. The median age at the time of diagnosis was 66 years (IQR: [55;76]) with a male predominance (59%). At diagnosis, 196 (48%) patients had high leukocyte counts (*ie* ≥ 11 G/L), 73 (18%) had a palpable splenomegaly and 94 (23%) and 84 (20%) patients had previous venous and arterial thrombosis, respectively. Three hundred twenty eight (75%), two hundred thirty seven (58%), and forty nine (11%) patients were classified as “high-risk” patients according to the ELN [4], IWG-PV [20] and MIPSS-PV scores [18], respectively. The most common first-line therapy was hydroxycarbamide (72%) followed by interferon (18%). A *JAK2* mutation was detected in all the patients, 430 of them (98%) carrying a *JAK2*V617F mutation and 9 patients (2%) having a *JAK2*-exon 12 mutation. The characteristics of the cohort are detailed in Supplementary Table S1.

### Molecular landscape of additional mutations in PV

**Fig. 1:**
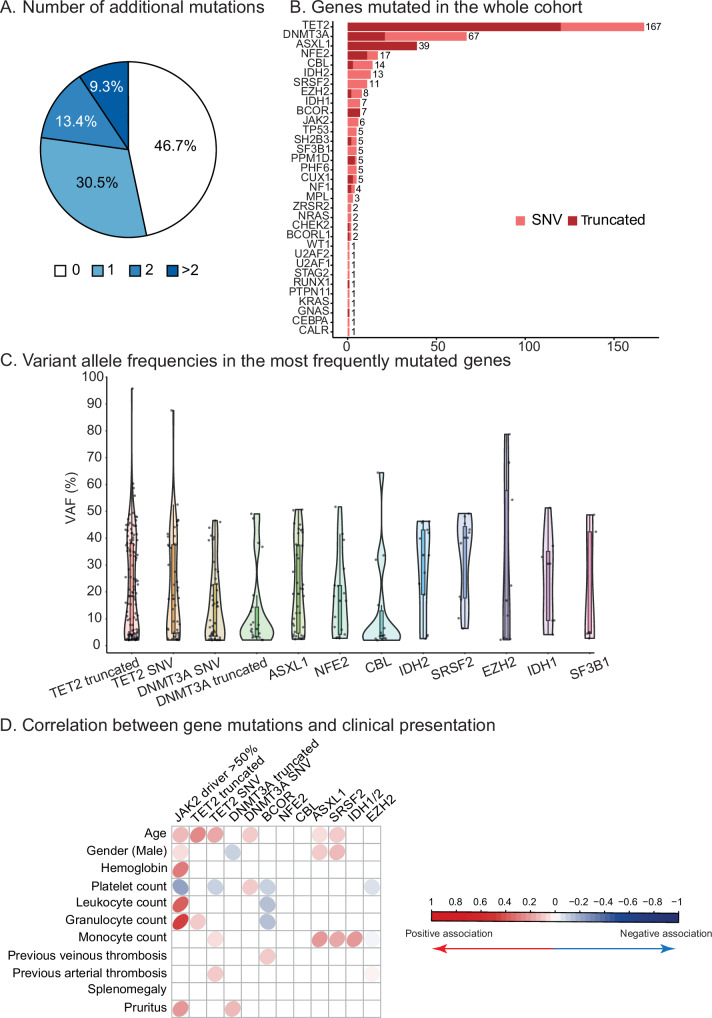
Mutational landscape of the whole cohort. **A** Distribution of the number of additional mutations (*i.e*. not *JAK2*-driver) in the cohort of 439 PV patients. **B** Total number of mutations per genes according to the type of mutation: truncated for nonsense or frameshift and SNV for others. **C** The distribution of allele burden of additional mutations was represented by violin plots. **D** Correlation plot showing the positive and negative association between mutations and clinical or biological presentation at the time of diagnosis. Only associations with a *p*-value < 0.05 are reported.

To explore the contribution of molecular characterization in improving the prognostic stratification of PV patients, we used a targeted sequencing strategy to search for additional mutations in a panel of 36 genes. While 205 patients (46.7%) had only a canonical *JAK2* mutation, we detected at least one additional mutation in 234 patients (53.3%, Fig. 1A). Patients with multiple additional mutations were not rare with 13.4% and 9.3% of patients harboring 2 and more additional mutations, respectively (Fig. 1A). As described previously [17, 18], the 411 pathogenic or likely pathogenic mutations detected most frequently involved the *TET2*, *DNMT3A* and *ASXL1* genes (Fig. 1B). While *TET2*, *ASXL1*, *NFE2* and *PPM1D* were mostly truncating mutations, *DNMT3A*, *CBL*, *SRSF2*, *IDH1/2*, *SF3B1* and *TP53* were mostly missense mutations. The variant allele frequency (VAF) distribution was variable according to the mutated gene. *DNMT3A* and *NFE2* mutations had a low VAF in most cases ( <20%), whereas *IDH2* and *SRSF2* were frequently present with a higher VAF ( ≥20%, Fig. 1C). VAF in other genes such as *TET2* and *ASXL1* had a bimodal distribution with a group of low VAF mutations and a group of VAF around 40%.

Beyond somatic mutations, it is well known that PV patients can acquire chromosomal alterations. The most frequently detected abnormality was the chromosome 9p uniparental disomy (9pUPD), observed in 45% (181/399) of patients. The detection of 9pUPD was correlated with higher VAF of *JAK2*V617F mutation and the presence of *TET2* mutations. Other CNV found were 9p trisomy (8 patients, 1.8%), del(20q) (3 patients, 0.7%), chromosome 7 abnormalities (one del7q and one 7qUPD, 0.5%), chromosome 1q gain (2 patients, 0.5%), partial del(13q) (1 patient, 0.2%) and chromosome 8 trisomy (1 patient, 0.2%). Excluding chromosome 9 abnormalities, a CNV was found in 1.6% of patients.

### Association of mutations with the clinico-biological presentation at diagnosis

After describing the genetic landscape of PV patients, we next explored potential associations between the molecular abnormalities and the presentation at diagnosis. We first focused on the most studied molecular parameter in MPN: *JAK2*V617F allele burden. The mean *JAK2*V617F allele burden was 37.6% (min 1.3%, max 98%). As previously shown, a *JAK2*V617F allele burden ≥50% (or a 9pUPD) was associated with higher hemoglobin, leukocyte and granulocyte counts, but lower platelet counts (Fig. 1D) [21–23]. In our cohort, *JAK2*V617F allele burden ≥50% was also associated with older age and male sex. Older age was also associated with more frequent *TET2*, *DNMT3A* missense, *ASXL1* and *SRSF2* mutations. Moreover, *BCOR* mutations were associated with lower platelet, leukocyte and granulocyte counts, whereas *ASXL1*, *SRSF2* and *IDH1/2* mutations were associated with higher monocyte counts (Fig. 1D). Only *TET2* missense mutations were significantly associated with prior arterial thrombosis, while *BCOR* mutations were significantly associated with prior venous thrombosis. (Fig. 1D). Finaly, *JAK2*V617F allele burden ≥50% and *DNMT3A* truncating mutations were associated with pruritus at diagnosis (Fig. 1D).

### Bayesian network analysis reveals the structure of molecular landscape in PV

**Fig. 2:**
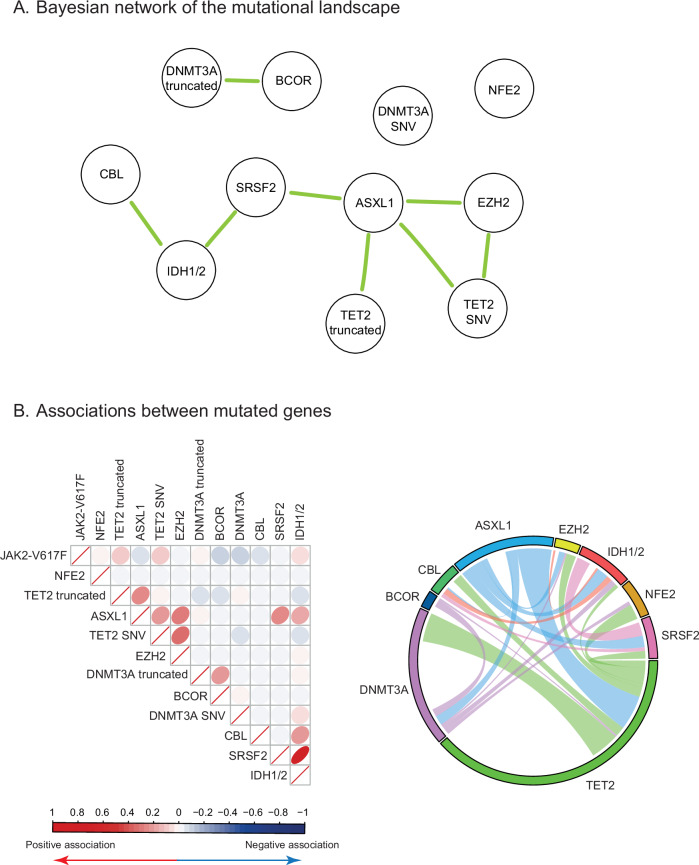
Mutational network derived from associations of mutations. **A** Bayesian network representing association between genes mutated, based on the allele frequencies and co-occurrence of mutations. Genes mutated in at least 7 patients were kept in the analysis. Green links represent a positive association. **B** Pairwise associations between mutated genes were represented by a correlation plot based on the allele burden of mutations (left panel) and a circos plot based on the presence of mutations (right panel). For the circos plot, truncating and SNV mutations were grouped for *TET2* and *DNMT3A* genes.

We next explored preferential associations between additional mutations using correlations and a Bayesian network analysis. Most notably, we observed that *ASXL1* mutations were a central node in the molecular landscape, with frequent association with other mutations in *TET2*, *EZH2*, *SRSF2* or *IDH1/2* genes (Fig. 2A, B). Thus, *ASXL1* mutations were isolated (*i.e*. without any other additional mutation) in only 22% of cases (8/36, Fig. 2B right panel). Moreover, we observed a significant association between *TET2* and *EZH2*, *DNMT3A* and *BCOR* as well as between *IDH1/2*, *CBL* and *SRSF2* mutations (Fig. 2A, B). Multiple *TET2* mutations were relatively frequent and found in 28 patients (6.4%, *data not shown*). Altogether, our results suggest that additional mutations in PV patients are associated in a non-random fashion and that different groups of patients can be distinguished based on their mutational landscape.

### Relationship between additional mutations and outcomes to define prognostic molecular groups

**Fig. 3:**
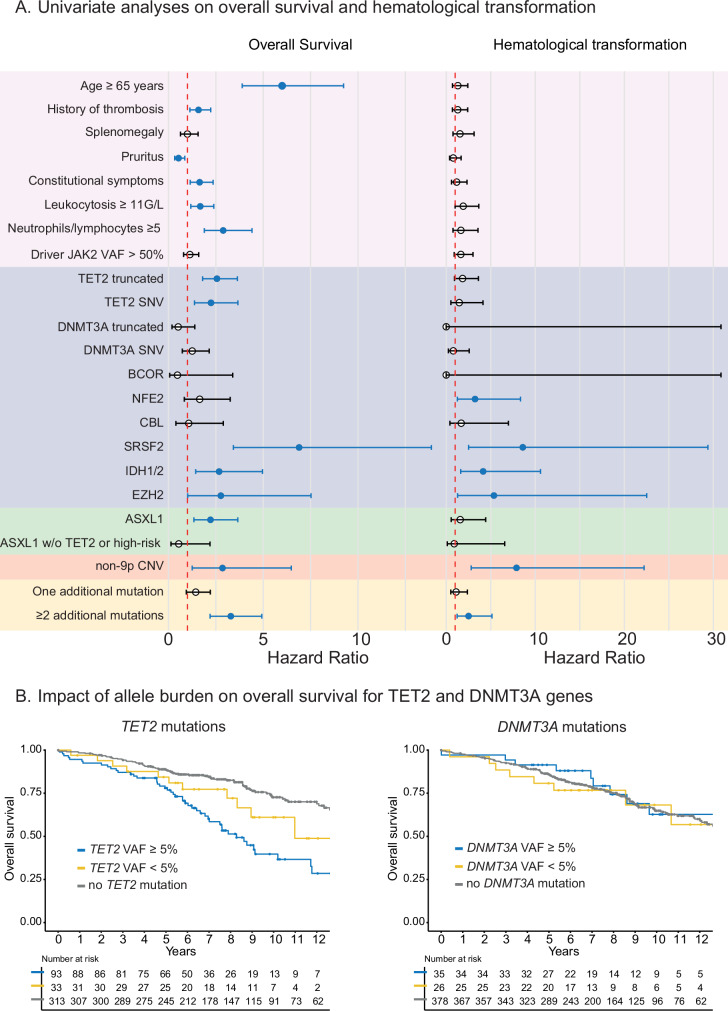
Prognostic impact of additional mutations. **A** Forest plots summarizing the individual impact of each genomic category for overall survival (left panel) and hematologic transformation (right panel). High risk mutations include *SRSF2*, *EZH2*, *IDH1*, *IDH2*, *CBL* and *NFE2* mutations. CNV include abnormalities of chromosomes 1q, 5, 7, 8, 13 and 20. Significant associations are represented in blue. **B** Kaplan-Meier curves showing the impact of allele burden on overall survival for *TET2* (left panel) and *DNMT3A* (right panel) mutations.

To define molecular groups with a homogeneous prognostic association, we next searched for a potential association between additional mutations and complications. With a median follow up of 7.8 years, 136 patients (31%) died, 31 (7.1%) evolved toward secondary myelofibrosis and 14 (3.2%) transformed to myelodysplastic syndrome/acute myeloid leukemia (MDS/AML). The cause of death was known in 52% of patients (70/132) and was related to the disease in 64% of cases (13 leukemic transformation, 11 thrombosis and 21 due to cytopenia). Univariate analysis for overall survival and hematologic transformation are summarized by forest plots in Fig. 3A. Among classical prognostic factors described in PV, older age, history of thrombosis, constitutional symptoms (fatigue, sweating or weight loss), leukocytosis ≥ 11 G/L and neutrophil/lymphocyte ratio ≥5 were associated with decreased overall survival, but not with risk of hematologic transformation. Among molecular markers, *TET2* missense (HR: 2.24 [1.38–3.66]), *TET2* truncating (HR: 2.56 [1.80–3.64]), *SRSF2* (HR: 6.89 [3.43–13.87]), *IDH1/2* (HR: 2.67 [1.44–4.96]), *EZH2* (HR: 2.77 [1.02–7.52]) and *ASXL1* (HR: 2.22 [1.35–3.66]) mutations were associated with decreased overall survival (OS, Fig. 3A). Similarly, *NFE2* (HR: 3.24 [1.26–8.34]), *SRSF2* (HR: 8.60 [2.52-29.4]), *IDH1/2* (HR: 4.14 [1.62–10.6]) and *EZH2* (HR: 5.35 [1.27–22.5]) mutations were associated with decreased transformation-free survival (TFS, Fig. 3A). In more details, *NFE2*, *SRSF2* and *EZH2* were associated with secondary myelofibrosis, while *SRSF2* and *IDH1/2* were associated with AML/MDS transformations (Supplementary Fig. S1). Interestingly, the association between *ASXL1* mutations and reduced OS was no longer observed when they were not associated with *TET2* or other “high-risk” mutations (HR: 0.90 [0.12–6.57]) (Fig. 3A). Because 22.7% of our PV patients had multiple mutations (*i.e*. ≥ 2), we also evaluated the prognostic association of the total number of additional mutations detected in a single patient. We observed a negative impact of this parameter on both OS (HR: 3.29 [2.20–4.93]) and TFS (HR: 2.51 [1.22–5.14]) (Fig. 3A). Finally, we also observed that detecting a non-9p CNV was associated with decreased OS (HR: 2.85 [1.26–6.48]) and TFS (HR: 7.89 [2.80–22.2]) (Fig. 3A).

Because *TET2* and *DNMT3A* mutations were significantly associated with age, we performed a sensitivity analysis using different VAF thresholds to identify a specific association with survival and exclude a potential effect mediated by age-related clonal hematopoiesis. While *DNMT3A* mutations were not associated with outcomes, *TET2* mutations were associated with a reduced survival (HR:2.64 [1.85–3.77], *p* < 0.001) and an increased risk of hematologic transformation (HR:2.25 [1.16–4.35], *p* = 0.016) when present with a VAF ≥ 5% or higher (Fig. 3B).

Finally, we also evaluated the association between genetic alterations and the risk of incident thrombosis. While an age older than 65 years (HR: 3.27[1.29–8.45]), arterial hypertension (HR: 2.72 [1.11–6.67]), and *NFE2* mutations (HR: 6.39 [2.16–18.90]) were associated with the occurrence of arterial thrombosis during follow up, only prior venous thrombosis (HR: 3.70 [2.11–6.48]) was associated with venous thrombotic events during follow-up (Supplementary Fig. S2).

Altogether, our results suggest that *SRSF2*, *IDH1/2*, *EZH2* and *NFE2* mutations and at a lesser extent *TET2* mutations represent high-risk mutations in PV patients. Moreover, the detection of 2 or more additional mutations or a non-9p CNV can also be considered as poor prognosis markers in PV patients.

### Development of a molecular prognostic signature

**Fig. 4:**
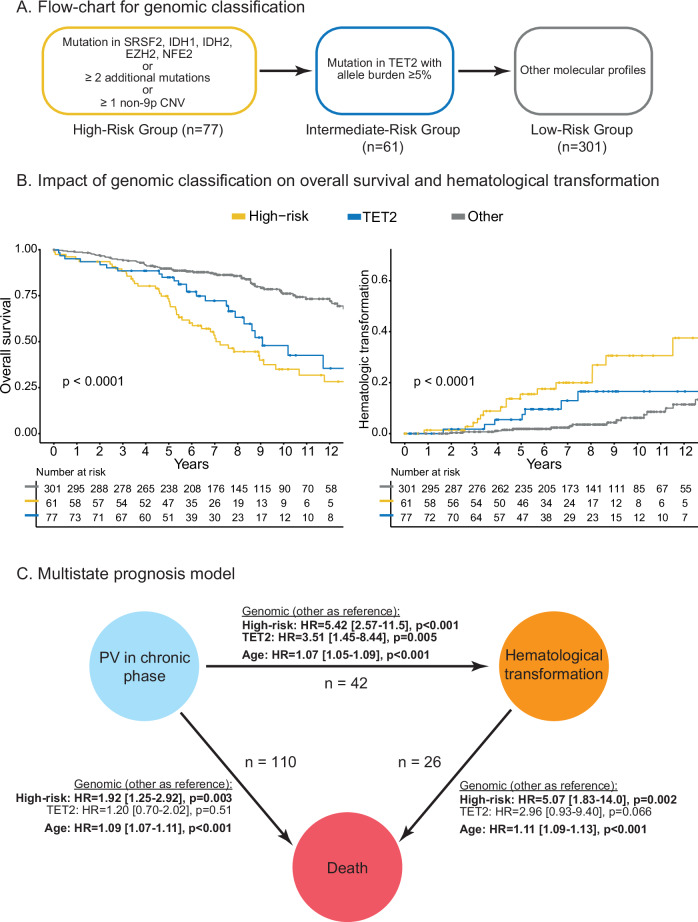
A genomic classification for predicting survival and transformation. **A** Sequential definition of the three genomic groups. **B** Kaplan-Meier curves showing the impact of the genomic groups on overall survival (left panel) and hematologic transformation (right panel). **C** Results of the multistate model considering the transitions between chronic phase, hematologic transformations and death. The following variables at diagnosis were included: genomic groups, age, gender, history of thrombosis, leukocytes and platelets counts, neutrophil/lymphocyte ratio and constitutional symptoms. A stepwise downward selection was performed. PV polycythemia vera.

Based on the bayesian network of molecular landscape and individual prognostic impact of gene mutations, we defined a ‘PV-HMR’ (High-Molecular-Risk) signature as patients harboring mutations in *SRSF2*, *IDH1/2*, *EZH2* and/or *NFE2*, or more than one additional mutation or presence of at least one non-9p CNV. Patients with *TET2* mutations with a VAF ≥ 5% were considered at “intermediate risk” while other genetic profiles were considered at “low risk” (Fig. 4A). In univariate analysis, these genetic categories efficiently predicted both OS and TFS (Fig. 4B).

In order to determine whether this “PV-HMR” signature provides additional information to clinical and biological data to predict OS and TFS in PV patients, we used a multistate modeling, which allows to analyze each transition between chronic phase, hematological transformation and death using multivariate cox models. The following variables were included in each model: age at diagnosis, gender, history of thrombosis, constitutional symptoms, leukocyte count, platelet count, neutrophil/lymphocyte ratio and the 3-tier genomic classification. The final model is summarized in Fig. 4C. PV-HMR groups and age at diagnosis were associated with an independent and significantly higher risk of hematologic transformation (HR of 5.42 [2.57–11.5], *P* < 0.001 and 1.07 [1.05–1.09], *P* < 0.001, respectively) and risk of death without hematologic transformation (HR of 1.92 [1.25–2.92], *P* = 0.003 and 1.09 [1.07–1.11], *P* < 0.001, respectively). Intermediate-risk group was associated with an increased risk of hematologic transformation (HR of 3.51 [1.45–8.44], *P* = 0.005), but not with the risk of death because of an older age at diagnosis (Supplemental Table S2).

### Validation of the prognostic classification and comparison of performances

**Fig. 5:**
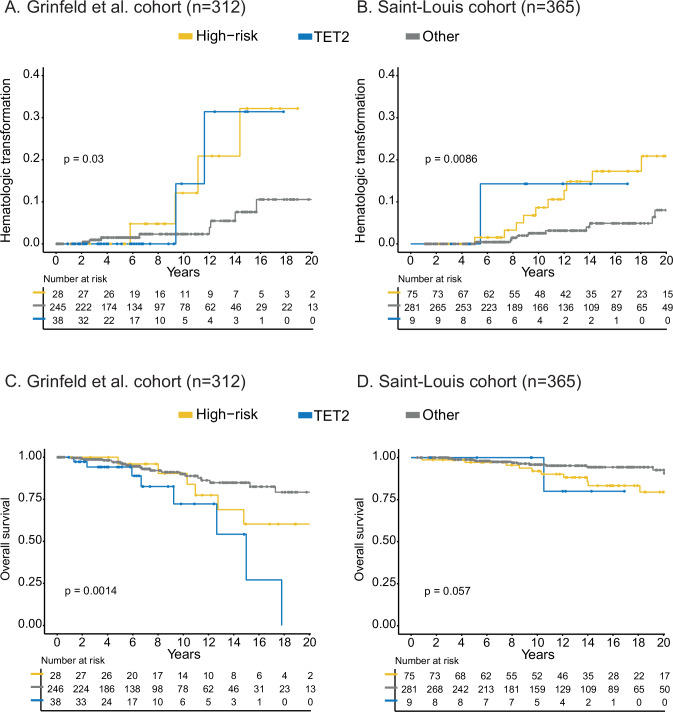
Validation of the molecular signature impact in two external cohorts. Kaplan-Meier curves showing hematologic transformation in **A** Grinfeld et al. cohort and **B** Saint-Louis Hospital cohort, and overall survival in **C** Grinfeld et al. cohort and **D** Saint-Louis Hospital cohort.

Finally, we applied our PV-HMR signature to 2 independent external cohorts of 316 and 365 PV patients, from the data published by Grinfeld et al. [19] (median age of 61.5 years and 55% males) and from St-Louis Hospital (median age of 52 years and 55% males). In univariate analysis, we found that our genomic classification efficiently predicted the risk of hematologic transformation in these 2 external cohorts (Fig. 5A, B). The classification also identified patients with reduced OS in both cohorts, although the survival dynamics were different in the St-Louis cohort due to their younger age at diagnosis (Fig. 5C, D).

We then aimed to evaluate the added value of our new PV-HMR signature combined with age for prognostic assessment as compared to other standard prognostic classifications (*i.e*., IWG-PV and MIPSS-PV). For this purpose, the performance (C-index and area under the curve (AUC)) [24, 25] and the accuracy (Brier score) [26] for predicting death or hematologic transformation were evaluated. The results are summarized in Table 1. For overall survival prediction, the MIPSS-PV scoring system performed better for early deaths (*i.e*. at 6 years), but our model combining the 3-tier genomic classification with age at diagnosis improved accuracy in predicting death at 10 or 14 years of follow-up. Regarding hematologic transformation, our model had the best overall performances in the 3 cohorts with the highest C-index in our and St-Louis cohorts and the highest AUC in our and Grinfeld et al. cohorts.

**Table 1:** Prognostic performance comparison.

Overall survival		C-Index	Events at 6 years		Events at 10 years		Events at 14 years	
			Brier score	AUC	Brier score	AUC	Brier score	AUC
**FIM cohort** ***n*** = **439**	PV-HRM+age	**0.83**	**0.057**	**0.855**	**0.080**	**0.907**	**0.093**	**0.892**
	IWG-PV	0.71	0.064	0.722	0.098	0.808	0.114	0.839
	MIPSS-PV	0.74	0.065	0.778	0.095	0.804	0.117	0.795
**Grinfeld et al.** ***n*** = **312**	PV-HRM+age	**0.81**	0.018	0.796	**0.038**	**0.846**	**0.057**	**0.878**
	IWG-PV	0.74	0.019	0.787	0.041	0.834	0.065	0.874
	MIPSS-PV	0.75	**0.017**	**0.820**	0.039	0.735	0.064	0.736
**St Louis, Paris** ***n*** = **365**	PV-HRM+age	**0.68**	0.009	0.509	0.018	0.625	0.029	0.765
	IWG-PV	0.60	0.013	0.469	0.027	**0.657**	0.041	**0.847**
	MIPSS-PV	0.66	**0.008**	**0.562**	0.018	0.634	**0.028**	0.754
Hematologic transformations		**C-Index**	**Events at 6 years**		**Events at 10 years**		**Events at 14 years**	
			Brier score	AUC	Brier score	AUC	Brier score	AUC
**FIM cohort** ***n*** = **439**	PV-HRM+age	**0.75**	**0.019**	**0.799**	**0.039**	**0.794**	**0.062**	**0.746**
	IWG-PV	0.63	0.021	0.643	0.041	0.710	0.066	0.707
	MIPSS-PV	0.61	0.020	0.646	0.042	0.636	0.066	0.689
**Grinfeld et al.** ***n*** = **312**	PV-HRM+age	0.54	0.006	**0.488**	0.014	**0.747**	**0.027**	**0.7376**
	IWG-PV	**0.61**	0.007	0.470	0.014	0.744	0.030	0.727
	MIPSS-PV	0.53	0.006	0.408	0.014	0.594	0.028	0.610
**St Louis, Paris** ***n*** = **365**	PV-HRM+age	**0.66**	0.001	0.735	0.009	**0.666**	0.021	0.664
	IWG-PV	0.60	0.002	**0.737**	0.012	0.547	0.027	**0.677**
	MIPSS-PV	0.59	0.001	0.609	0.009	0.588	0.021	0.625

C-index and AUC evaluated the performance of the model and Brier score reflected the accuracy of prediction (i.e. the rate of error). The best values for each cohort and event were in bold.

## Discussion

Over the past years, genetic analyses have been increasingly used to refine the prognostic stratification of patients with hematologic malignancies. Due to a higher number of mutations, and a more aggressive course, PMF patient management has rapidly benefitted from molecular scoring systems. Thus, “high-molecular risk” mutations in *ASXL1*, *EZH2*, *SRSF2*, *IDH1/2* and *U2AF1* genes [12, 15] have been used to develop genetic-based scoring systems [13–15] that are now widely used in clinical practice to guide patient management. In PV patients, molecular risk scores have not yet been included in practical patient management, probably because of the low number of studies which have searched for a prognostic impact of additional mutations in these patients, as well as the rather low number of PV patients included in previous cohorts.

As previously observed [10, 17, 27, 28], we detected an additional, non-driver mutation in more than a half of our patients, with mutations in *DNMT3A*, *TET2* and *ASXL1* being the most frequent. These “DTA” mutations were associated with older age at diagnosis as previously shown [28, 29], which is consistent with their frequent detection in age-related clonal hematopoiesis of indeterminate potential [30, 31]. Notably, 22.7% of our patients had 2 or more mutations, a proportion that also aligns with previous studies [17, 28]. Interestingly, we observed that the association between additional mutations was not random. Using a bayesian approach, we were able to discriminate homogeneous groups of mutational profiles. *NFE2*- and *BCOR/DNMT3A*-mutated patients were identified as distinct groups of patients, while *ASXL1* mutations were very frequently associated with other mutations, especially in other epigenetic regulators and splicing factors. Noteworthy, this network showed characteristics similar to this developed by Grinfeld et al. on a mixed cohort of ET, PV and PMF patients [19].

The main aim of our study was to identify mutational profiles that could discriminate patients with a higher risk of hematologic evolution. Based on the Bayesian network and the impact of individual mutations on the disease progression, we defined a new high molecular risk signature (PV-HMR) associated with a decreased OS and more importantly TFS. This signature includes single gene-mutations in *SRSF2*, *EZH2*, *IDH1*, *IDH2* or *NFE2*, a total number of somatic, non-driver mutations ≥2 and/or non-9p CNV. Among these, only *SRSF2* mutations were included in the MIPSS-PV scoring system [18]. This is concordant with the observation that, in our cohort, *SRSF2* mutations were associated with the highest risk of both death and hematologic progression. However, studying a large number of patients, we were able to identify other somatic mutations associated with an adverse prognosis. Interestingly, *EZH2*, *IDH1*, *IDH2* mutations are also included in the prognostic scores in PMF [12, 13], and *NFE2* mutations were previously reported to be associated with a decreased transformation-free survival in a cohort of MPN patients [32]. In our PV patients, *ASXL1* mutations were associated with a decreased OS, but this effect was mainly driven by their association with other “high-risk” mutations since patients with isolated *ASXL1* mutation did not have decreased overall or transformation-free survival. This finding is consistent with the lack of prognostic impact per se of *ASXL1* mutations in PMF previously reported [16, 33] and reinforces the importance of considering the associations between mutations for prognostic assessment. Beyond the effect of specific mutations, we observed that carrying 2 or more mutations was also associated with an increased risk of death or transformation. Lundberg et al. also reported a detrimental effect of having a high number of additional mutations in MPN patients [10], which may be the reflect of a greater genetic instability.

More surprisingly, *TET2* mutations with an allele burden ≥5% were associated with an intermediate risk of hematologic progression between the PV-HMR group and other patients. An adverse prognosis of *TET2* mutations has already been suggested in the study by Lundberg et al. [10], but was not found in other studies. *TET2* mutations can be acquired early, before the driver, or secondary to the driver mutation, and this order of acquisition seems to influence the phenotype and course of the disease [34]. Determining the clonal architecture in future studies may help to refine the prognostic role of *TET2* mutations in MPN. Finally, we found that non-9p CNV was also a poor-prognosis marker, which is consistent with previous studies showing an adverse prognostic impact of abnormal conventional cytogenetics [35, 36]. Of note, the sensitivity and specificity between CNV detection by NGS assay on mature blood cells and conventional karyotype are different, and the results cannot be directly compared.

Applying our 3-tier molecular signature (PV-HMR, *TET2* and others) in 2 external cohorts, we confirmed that we were able to identify patients at higher risk of hematologic progression. For OS, while the prediction was reproduced in the cohort of Grinfield et al., the lower performance observed in the Saint-Louis cohort was probably due to demographic differences, as median age at diagnosis in this cohort was lower than in ours (median ages 58 *vs.* 66 years). This could also reflect the lower capacity of genomic analysis to predict the risk of death compared to the risk of transformation, as suggested by Grinfeld et al. [19]. To demonstrate that this mutational signature could help to refine the prognostic stratification of PV patients beyond previously identified risk factors, we integrated our genomic classification together with clinical and biological data into a multistate model. We observed that the association of the PV-HMR signature with age was able to discriminate high-risk patients. Of note, although MIPSS-PV outperformed our model in predicting overall survival at 6 years, PV-HMR combined with age outperformed the existing scoring systems available for PV patients (IWG-PV score and MIPSS-PV) in predicting overall survival at 10 and 14 years and, most importantly, the risk of transformation at 6, 10 and 14 years. It will be of major interest to study whether relatively novel therapeutic approaches (like interferon alpha or JAK2-inhibitors) would be more beneficial in patients with higher transformation risks.

Our work has several limitations. Due to the retrospective nature of this work, some data were missing for a significant number of patients, particularly cause of death which was unavailable for 48% of subjects (although hematologic evolution can be excluded in these cases). In addition, we did not have sufficient data to evaluate the impact of treatment on patient outcomes. However, it should be noted that there was no difference in first-line cytoreductive drugs between patients belonging to the different prognostic groups of our genomic classification. Then, we did not evaluate the mutational profile of our patients on sequential samples which could be interesting to identify the emergence or appearance of clones that could have contributed to hematologic transformation. Finally, it would be interesting to study more patients to further refine our model and be able to predict the different types of hematologic transformation as Grinfeld et al. did.

However, our study also has several strengths. First, it represents the largest cohort of PV patients with centrally generated sequencing data, which allowed us to identify several associations between additional mutations and clinico-biological features of PV patients, but more importantly, to identify a relatively high number of genetic events associated with poor prognosis. In addition, we adopted an statistical approach to avoid some biases of the “classical analysis”. First, a Bayesian network was developed to consider the preferential associations between somatic mutations, and then, we chose to use a multistate model, which has the advantage of being able to evaluate transitions between the different phases of the disease.

In conclusion, we identified molecular abnormalities that identify PV patients with an increased mortality and increased risk of hematologic transformation. These results support the incorporation of additional mutations for the prognostic stratification of PV, in particular the identification of patients at high risk of hematologic progression who are not stratified by the current prognostic scoring systems. Validation in additional cohorts will be necessary to integrate its use in clinical practice, especially with the development of new targeted therapies in MPN.

## Supplementary information

Supplemental data

## Data Availability

The data that support the findings of this study are available on request from the corresponding author
