# Supplementary material for: A molecular signature predicts hematologic evolution in polycythemia vera patients
Source: Leukemia. 2025 Jun 18;39(8):1937–47. doi: 10.1038/s41375-025-02660-0 (PMC12310540; doi:10.1038/s41375-025-02660-0)
Supplement: Supplementary file 1 — Supplemental data [file 41375_2025_2660_MOESM1_ESM.pdf]

# Supplemental Data

## Selection of patients

Patients were recruited from the French Intergroup of Myeloproliferative Neoplasms (FIM) national database (BCB FIMBANK) if they received a diagnosis of PV between 2005 and 2018 according to the World Health Organization 2008 or 2016 classifications. The BCB FIMBANK has been approved by the Advisory Committee on Processing Information Associated with Health Research (Comité Consultatif sur le Traitement de l'Information en Matière de Recherche dans le Domaine de la Santé) on May 19, 2016 and underwent a standard declaration to the French Data Protection Authority (Commission Nationale de l'Informatique et des Libertés) on January 19, 2018.

Four hundred seventy one patients matched these criteria and their DNA were centralized. Diagnosis criteria were reviewed by clinicians, biologists and pathologists from the FIM group. This led to the exclusion of 13 patients.

***Reason for patient exclusion***

32 of the 471 patients/samples were excluded for the following reasons:

- Diagnosis before 2005 (n=8)
- Diagnosis of ET (n=1)
- Diagnosis of post-ET PV (n=1)
- Diagnosis reclassified as myelofibrosis because of biological presentation and rapid evolution to patent myelofibrosis (no bone-marrow biopsy at diagnosis for n=2 and MF2 fibrosis on BMB for n=1)
- Poor quality of DNA and/or NGS data (n=19)

***Center of origin of selected patients***

A total of 439 patients/samples was analyzed with the following repartition by French hospital of origin: CHU Angers (n=102), CHU Brest (n=72), CHU Nancy (n=68), CHU Dijon (n=58), CHU Bordeaux (n=55), APHP Créteil (n=18), CHU Toulouse (n=15), CH Rochefort (n=9), HCL Lyon (n=9), CH Cholet (n=9), CH Périgeux (n=8), CH Perpignan (n=6), CH Vannes (n=4), CH Annecy (n=4) and APHP Kremlin-Bicêtre (n=2).

## NGS Sequencing and Analysis

***Design***

A custom RNA-bait pool was designed in order to cover all coding exons of the 36 genes of interest (listed below).

| | | | |
|---|---|---|---|
| *JAK2* | *DNMT3A* | *SRSF2* | *NFE2* |
| *CALR* | *TET2* | *SF3B1* | *TP53* |
| *MPL* | *IDH1* | *U2AF1* | *GATA2* |
| *SH2B3* | *IDH2* | *ZRSR2* | *DDX41* |
| *CBL* | *ASXL1* | *CUX1* | *PPM1D* |
| *NF1* | *EZH2* | *SETBP1* | *PTPN11* |
| *NRAS* | *BCOR* | *STAG2* | *GNAS* |
| *KRAS* | *BCORL1* | *PHF6* | |
| *KIT* | *FLT3* | *CEBPa* | |
| *CSF3R* | *NPM1* | | |

Additional probes were added to study copy number variations (CNV) on chromosomes 1q, 5, 7, 8, 9p, 13q, 17p and 20q with a density of one probe every 5 Mbp. 9pUPD was not evaluable in 40 patients because of noisy NGS signal and higher variations of SNP's VAFs.

***Library preparation and sequencing***

DNA was quantified with QubitTM dsDNA BR Assay Kit. We used the SureSelectQXT Target kit from Agilent to build the libraries according to the manufacturer's recommendations. Approximately 50ng of DNA for each patient was randomly fragmented with enzymes and

adaptor-tagged in the first step. The DNA library was then amplified by PCR, and the amplicons purified with AMPure XP Beads, Beckman Coulter. DNA library quality was assessed using the Agilent 2100 Bioanalyzer and Agilent DNA 1000 Assay, and quantified with the QubitTM dsDNA HS Assay Kit. Between 500 and 750ng of the amplicons was hybridized using the SureSelect Capture Library and then captured on streptavidin-coated beads, to obtain an enriched targeted DNA-library. The library was PCR-amplified by using Dual Indexing primers, and purified with AMPure XP Beads, Beckman Coulter. Finally, libraries were pooled and sequenced on a NextSeq500 (Illumina) 2x150bp for a theorical median coverage of 3000X.

***Bioinformatic pipeline***

- Workflow management, pipeline availability

The analysis pipeline used in this study (FoxNGS-PV) is branched from our analysis pipeline used in routine, FoxNGS-H. It is a NextFlow pipeline (version 23.04.2.5870, openjdk 11.0.24). Each step described below is contained in a specific NextFlow process. Each process runs in a Singularity image, or a conda environment for processes running Python. Singularity images were fetched from Dockerhub, or designed using a custom .def file when necessary.

Version control is done using Git, with a remote public repository hosted on GitLab (gitlab.com/bioinfodiagangers/somatic/haematology/FoxNGS-H/-/tree/PV). This repository contains the pipeline code, code for all processes described below, singularity image .def files and DockerHub links.

- Pipeline quality assessment

The quality of both sequencing and bioinformatics were checked by analysis in each sequencing run of a commercial internal quality control with 22 mutations with VAF of 5 to 70% and long deletions/insertions (Horizon Myeloid DNA-HD829). A run was analyzed only if all the mutations of this control were detected. Furthermore, our lab participates every year in the

external quality evaluation from the French Group of Molecular Biology in Hematologic Malignancies (GBMHM). In routine, each process is individually tested with the nf-test framework (v0.8.1) using simulated data and expecting absolute identity of the results between each test using md5sum when applicable.

- Demultiplexing, alignment and post-alignment

Fastq files were produced from raw bcl files using bcl2fastq (v2.20.0.422) this specific step was performed before the pipeline run using a specific command. Each paired-end FASTQ file was aligned with bwa-mem (v0.7.17-r1188). Misaligned reads and reads outside of our capture coordinates were filtered using samtools v1.13 and bedtools v2.30.0, respectively. Duplicate reads were tagged, but not filtered, with the function MarkDuplicates from Picard v2.27.1. Base qualities were also recalibrated with the BaseRecalibrator/ApplyBQSR functions from GATK v4.2.6.1

- Sequence quality controls and coverage assessments.

Fastq file quality controls were performed with fastqc (v0.11.9). To check proper coverage of our regions of interest, .bam file coverage was assessed using mosdepth (v0.3.3). Any sample with a percentage of bases covered above 200x inferior to 95%, an on-target below 60% or a median coverage below 1000x went through further quality assessment, and was re-sequenced if necessary. MultiQC v1.13 produced a quality report in the form of a html file.

- Variant calling

Four different tools were used to detect variations with optimal accuracy: HaplotypeCaller and Mutect2 for state of the art, general purpose variant callers, Varscan2 for a high sensitivity, low specificity method and Pindel for long InDels.

Variant calling tools specifics and nonstandard parameters are detailed bellow:

- HaplotypeCaller and Mutect2 were overall used according to GATK best practices.
- Varscan (2.4.3) used a pileup as its input file. Samtools mpileup was used to produce this file, with specific parameters for high sensitivity.
- Pindel (0.2.5) was used with a bed file specifically targeting FLT3-ITD mutations and CALR driver InDels. We set the tool for a maximum InDel size of 500 bp.

  - CNV Analysis

CNV analysis was performed using CNVKit (v0.9.10), according to default parameters. 8 samples were used to form a pool of normal samples. Each sample was tested by cross validation, using the 7 other samples as the pool of normals. The target and antitarget average sizes were determined using the autobin function.

  - Annotation and filtering

Annovar (release 2020-06-08) was used for variant annotation. Each vcf file was annotated separately. The following databases were used for annotation:

- COSMIC 89 and 90 (Release 5 September 2019)
- gnomad 2.1.1 Genomes (Update, March 6, 2019)
- dbnsfp35a (release 2018-09-21): Score columns like (whole-exome SIFT, PolyPhen2 HDIV, PolyPhen2 HVAR, LRT, MutationTaster, MutationAssessor, FATHMM, PROVEAN, MetaSVM, MetaLR, VEST, M-CAP, CADD, GERP++, DANN, fathmm-MKL, Eigen, GenoCanyon, fitCons, PhyloP and SiPhy scores from dbNSFP) to classify impact of mutation
- clinvar (release 2019-03-05)
- cytoBand for chromosome coordinates
- IARC TP53 (release July 2019), a WHO database of TP53 mutations

The output is a csv file with a line for each variant and its annotations. The four .vcf files (one for each variant caller) were subsequently merged using a custom Python script. Variant quality filters were also applied in this script. Those filters are:

- Deep intronic and synonymous mutations were removed
- Variants with a VAF < 2% were removed
- Variants with a minor allele frequency (MAF) ≥ 1% in gnomAD were removed
- Variants known as recurring artifact were removed, using a manually curated local database

***Review and classification of mutations***

Finally, retained variants were reviewed by 2 molecular biologists from the French Intergroup of Myeloproliferative neoplasms (DLP and BC, EV, AC, AM, OM, EL or IS) for (i) visual inspection of reads in BAM file to conclude in vavour of a real mutation or an artifact and for (ii) classification of the pathogenicity of mutations. Cases of discrepancies were discussed in concertation meetings. The classification of mutations was based on the consensus recommendation of the Association for Molecular Pathology and the American Society of Clinical Oncology[6]. Variants were classified as pathogenic, likely pathogenic or of unknow significance according to the following criteria. Furthermore, we decided also to remove all mutations with a MAF ≥ 0.01% in the category of variant of unknown significance because they were probably rare polymorphisms.

***Classification of additional mutations***

| | |
|---|---|
| Pathogenic | - Mutation non-sens or frameshift<br>- Mutation described in myeloid malignancy (COSMIC) with somatic validation<br>- Mutation with a functional effect demonstrated |
| Likely Pathogenic | - Mutation not classified as pathogenic with a VAF <40% or >60% |
| Variant of Unknown Significance | - Mutation not classified as pathogenic or likely pathogenic with a VAF between 40 and 60 % |

## Statistical analysis

***Descriptive analysis***

Quantitative variables were reported as median and ranges and qualitative variables as proportions. Comparisons were performed using Mann and Whitney test for quantitative variables of Fisher test for qualitative variables. All p-values were corrected using a Benjamini-Hochberg procedure in order to control the False Discovery Rate.

***Bayesian network and identification of genomic subgroups***

A Bayesien Network (BN) was performed by first creating a binary matrix indicating the presence of mutation for genes mutated in at least 7 patients in the whole cohort (i.e. 2% of the population). Analysis was performed using *bnlearn* package under R software. In details, a forward selection technique for neighborhood detection based on the maximization of the minimum association measure observed with any subset of the nodes selected in the previous iterations. Hierarchical clustering analysis (HCA) was performed to create homogeneous groups based on the selected mutated genes in the BN. This analysis was performed using the Ward's method for linkage criteria combined with a Euclidean distance measure. Finally,

groups were determined using BN, HCA and, to be consistent with the available knowledge, with the BN described in *Grinfeld et al.* N=2035 patients).

***Multistate model***

This model aims to decipher the natural history of polycythemia vera and variable associated with each transition. Therefore, individual Cox proportional hazard models were performed for transitions from (i) PV chronic phase to hematological transformation to either acute myeloid leukemia, myelodysplastic syndrome or myelofibrosis, (ii) PV chronic phase to death and (iii) hematological transformation to death. Time zero was taken to be the time of diagnosis of PV. Observations were right-censored at the end of the follow-up.

Variables included in the model were:

Demographic:

- Gender (female as reference)
- Age at diagnosis, years (continue variable)

Clinical and biological features at diagnosis:

- History of thrombosis (no as reference)
- Constitutional symptoms (no as reference)
- Leukocyte count, $10^9$/L (continue variable)
- Platelet count, $10^9$/L (continue variable)
- Neutrophil / lymphocyte ratio ≥ 5 (NLR)

Molecular:

- Genomic groups defined as High-risk, *TET2* and Other (reference as Other)

A manual backward step by step selection was done except for the interest variable, i.e. genomics groups. Variance inflation factor (VIF) was computed to check the absence of collinearity against dependent variables. Scaled-Schonfeld residuals were computed to check the proportionality assumption. Statistical tests were performed to validate the non-proportionality combined with a plot of these residuals against time.

These individuals' transition models were used to perform a single multistate model, and death was the only state considered as terminal event. Analysis was performed using *mstate* package.

***Prognostic evaluation***

To evaluate the added value of genomic classification to classical prognostic tools (see section Clinical and Biological data), we computed the time-dependent AUC (area under receiving operating curve), Brier score and C-index. Indeed, the concordance of models was evaluated using C-index and AUC and the accuracy of the prediction was assessed using the Brier score (integrated Brier score and Brier score over-time). A graphical approach for time-dependent AUC and Brier score over-time was used to have a global evaluation of each prognostic tool. These analyses were performed with the following R packages: *pec, timeROC, pROC* and *survival*

**Supplemental table S1: characteristics of the cohort of 439 PV**

| Variable | Whole cohort (n=439) | Number of missing values |
|---|---|---|
| Age at diagnosis (years) | 66 [55-76] | 0 |
| Gender: male | 258 (59%) | 0 |
| Splenomegaly at diagnosis | 73 (18%) | 34 |
| Pruritus at diagnosis | 100 (25%) | 31 |
| History of thrombosis | | |
| Arterial | 84 (20%) | 0 |
| Venous | 94 (23%) | 0 |
| Increased LDH | 75 (29%) | 171 |
| Hemoglobin (g/dl) | | |
| Male | 18.1 [16.9-19.7] | 10 |
| Female | 17.5 [16.9-19.7] | 8 |
| Hematocrit (%) | | |
| Male | 55.2 [51.7-59.8] | 12 |
| Female | 54.4 [50.5-59.1] | 9 |
| Platelet count (10^9/l) | 478 [320-628] | 27 |
| Leukocyte count (10^9/l) | 10.7 [8.4-13.4] | 30 |
| Granulocyte count (10^9/l) | 8.2 [6-10.3] | 61 |
| ELN risk category | | |
| Low | 111 (25%) | - |
| High | 328 (75%) | |
| IWG-PV risk category | | |
| Low | 74 (18%) | 30 |
| Intermediate | 98 (24%) | |
| High | 237 (58%) | |
| MIPSS-PV risk category | | |
| Low | 209 (48%) | - |
| Intermediate | 181 (41%) | |
| High | 49 (11%) | |
| Events during follow-up | | |
| Deaths | 136 (31%) | - |
| Secondary myelofibrosis | 31 (7.1%) | |
| Myelodysplasia | 2 (0.5%) | |
| Acute myeloid leukemia | 12 (2.7%) | |
| Veinous thrombosis | 49 (11%) | |
| Arterial thrombosis | 22 (5%) | |
| First-line treatment | | |
| Hydroxycarbamide | 287 (72%) | 42 |
| Interferon | 71 (18%) | |
| Pipobroman | 8 (2%) | |
| Venesection only | 31 (8%) | |

*Quantitative variables were resumed as median and interquartile ranges and qualitative variables as number and frequency.*

**Supplemental table S2: characteristics of the three genomic categories**

| Variable | High-risk (n=77) | Intermediate-risk (n=61) i.e. *TET2*≥5% | Low-risk (n=301) | High risk vs Int. risk | High risk vs Low risk | Int. risk vs Low risk |
|---|---|---|---|---|---|---|
| Age at diagnosis (years) | 72 [62-81] | 70 [61-81] | 64 [52-74] | 0.704 | **<0.001** | **0.001** |
| Gender: male | 71.4% | 45.9% | 57.8% | **0.012** | 0.060 | 0.117 |
| Splenomegaly at diagnosis | 16.2% | 14.5% | 19.1% | 1 | 1 | 1 |
| Pruritus at diagnosis | 25% | 30.9% | 22.8% | 0.822 | 0.822 | 0.796 |
| History of thrombosis | | | | | | |
| Arterial | 22.1% | 21.3% | 17.9% | 1 | 0.993 | 0.993 |
| Venous | 24.7% | 13.1% | 21.9% | 0.250 | 0.717 | 0.250 |
| Platelet count (10^9/l) | 446 [262-569] | 400 [278-597] | 512 [350-655] | 0.818 | **0.023** | **0.049** |
| Leukocyte count (10^9/l) | 10.4 [8.15-14.0] | 11.8 [10.0-14.8] | 10.6 [8.28-13.0] | 0.279 | 0.636 | 0.064 |
| Granulocyte count (10^9/l) | 7.9 [5.45-11.3] | 8.5 [6.88-12.0] | 8.15 [5.97-9.90] | 0.320 | 0.718 | 0.126 |
| ELN risk category | | | | | | |
| Low | 11.7% | 19.7% | 29.9% | 0.290 | **0.006** | 0.215 |
| High | 88.3% | 80.3% | 70.1% | | | |
| IWG-PV risk category | | | | | | |
| Low | 8.5% | 10.3% | 22.1% | 0.664 | **<0.001** | **0.018** |
| Intermediate | 14.1% | 19% | 27.5% | | | |
| High | 77.5% | 70.7% | 50.4% | | | |
| MIPSS-PV risk category | | | | | | |
| Low | 33.8% | 31.1% | 54.5% | 0.285 | **<0.001** | **0.006** |
| Intermediate | 44.2% | 55.7% | 37.9% | | | |
| High | 22.1% | 13.1% | 7.64% | | | |
| First-line treatment | | | | | | |
| Hydroxycarbamide | 75.4% | 77.4% | 70.9% | | | |
| Interferon | 13% | 13.2% | 20% | 0.974 | 0.493 | 0.692 |
| Pipobroman | 1.4% | 1.9% | 1.8% | | | |
| Venesection only | 10.1% | 7.5% | 7.2% | | | |

*Quantitative variables were resumed as median and interquartile ranges and qualitative variables as frequency. Multiple testing p-values were corrected with the Benjamini & Hochberg method.*

**Supplemental Fig. S1: Forest plots representing univariate analysis for impact of genetic alterations on myelofibrosis (left panel) and AML/MDS (righ panel) evolutions**

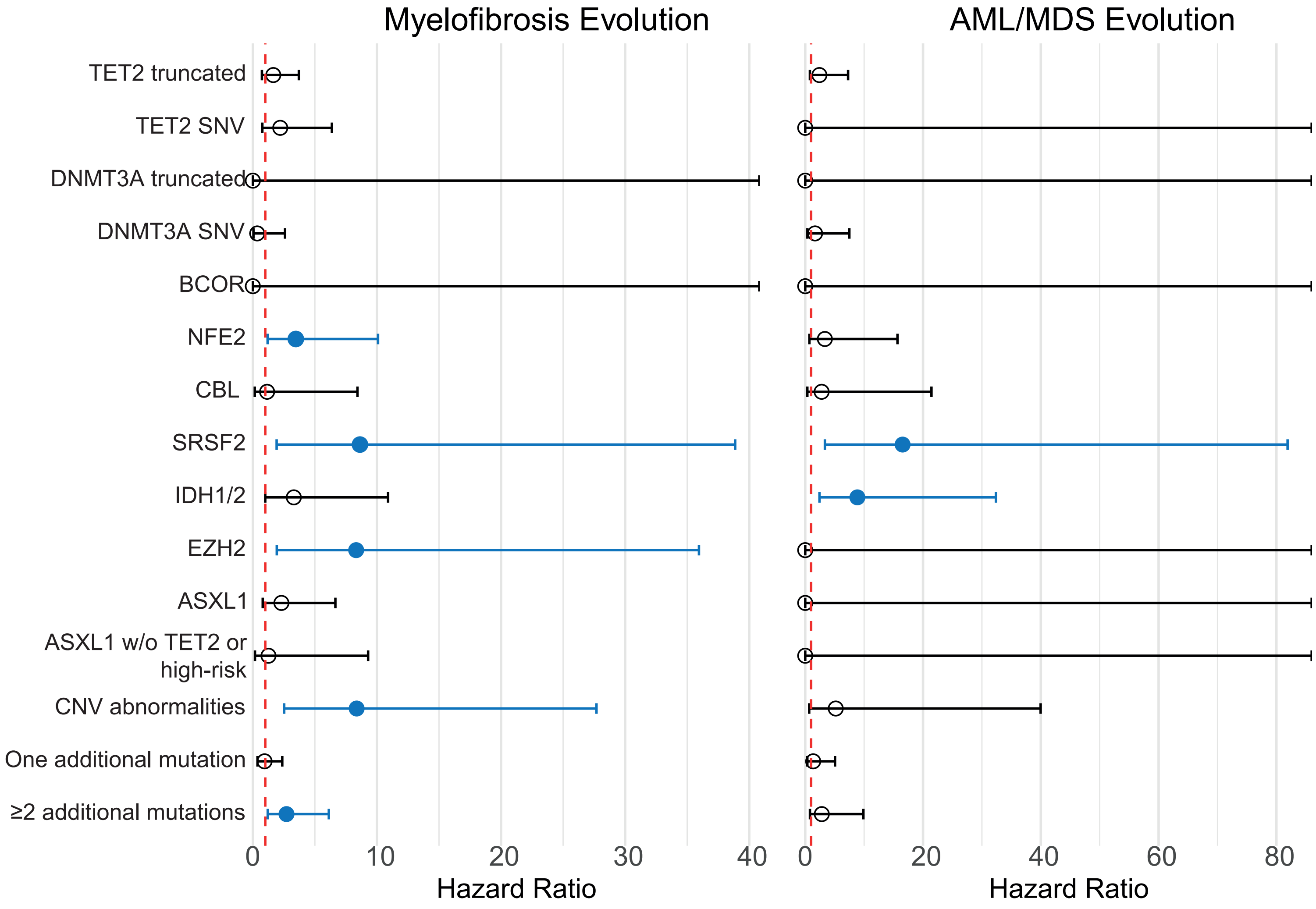


*Significant associations are in blue*

**Supplemental Fig. S2: Forest plots representing univariate analysis for variables associated with arterial (left panel) and venous (right panel) thrombotic events during follow-up**

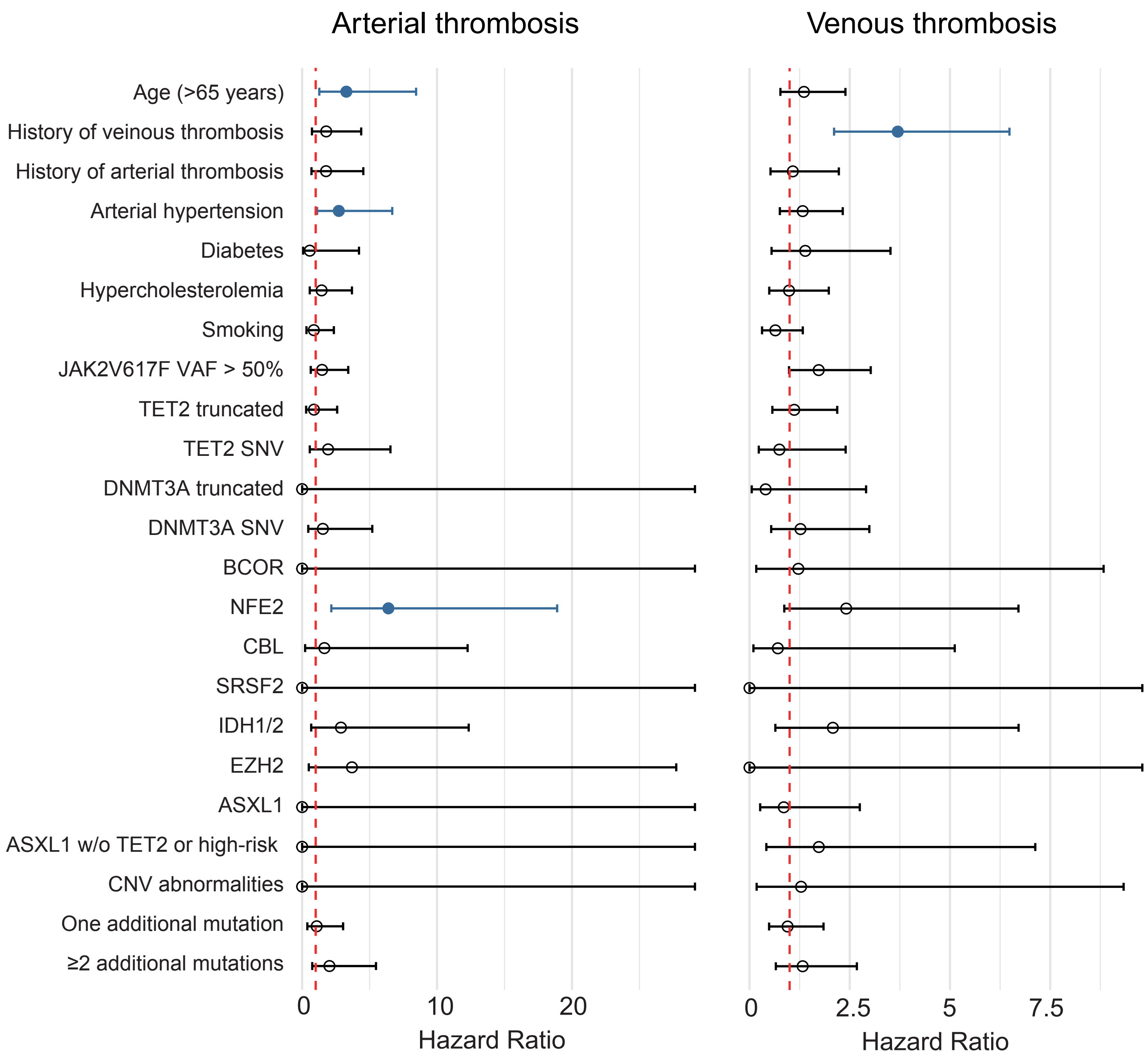


*Significant associations are in blue*

# Checklist for REporting of tumor MARKer Studies (REMARK) guidelines

| | |
|---|---|
| **Introduction**<br>1. State the marker examined, the study objectives, and any prespecified hypotheses. | Introduction section (page 4) |
| **Materials and Methods** | |
| **Patients** | |
| 2. Describe the characteristics (eg, disease stage or comorbidities) of the study patients, including their source and inclusion and exclusion criteria. | Supplemental Table SI |
| 3. Describe treatments received and how chosen (eg, randomized or rule-based). Specimen characteristics | Not applicable |
| 4. Describe the type of biological material used (including control samples) and methods of preservation and storage. | 'Patients and samples' part of the Method section (page 4-5) |
| **Assay methods** | |
| 5. Specify the assay method used and provide (or reference) a detailed protocol, including specific reagents or kits used, quality control procedures, reproducibility assessments, quantitation methods, and scoring and reporting protocols. Specify whether and how assays were performed blinded to the study end point. | Supplemental data |
| **Study design** | |
| 6. State the method of case selection, including whether the study design was prospective or retrospective and whether stratification or matching (eg, by stage of disease or age) was used. Specify the time period from which cases were taken, the end of the follow-up period, and the median follow-up time. | 'Patients and samples' part of the Method section (page 4-5) and 'selection of patients' section in Supplemental data |
| 7. Precisely define all clinical end points examined. | 'Statistics' section (page 6) |
| 8. List all candidate variables initially examined or considered for inclusion in models. | Page 8 of Supplemental data |
| 9. Give rationale for sample size; if the study was designed to detect a specified effect size, give the target power and effect size. | Not applicable |
| **Statistical analysis methods** | |
| 10. Specify all statistical methods, including details of any variable selection procedures and other model-building issues, how model assumptions were verified, and how missing data were handled. | Supplemental data for multistate model page8 |
| 11. Clarify how marker values were handled in the analyses; if relevant, describe methods used for cutpoint determination. | Not applicable |
| **Results** | |
| **Data** | |
| 12. Describe the flow of patients through the study, including the number of patients included in each stage of the analysis (a diagram may be helpful) and reasons for dropout. Specifically, both overall and for each subgroup extensively examined report the numbers of patients and the number of events. | Selection and exclusion of patients in Supplemental data Page 1 and Supplemental Table 1 |
| 13. Report distributions of basic demographic characteristics (at least age and sex), standard (disease-specific) prognostic variables, and tumor marker, including numbers of missing values. | Supplemental Table 1 |
| **Analysis and presentation** | |
| 14. Show the relation of the marker to standard prognostic variables. | Supplemental Table 2 |
| 15. Present univariate analyses showing the relation between the marker and outcome, with the estimated effect (eg, hazard ratio and survival probability). Preferably provide similar analyses for all other variables being analyzed. For the effect of a tumor marker on a time-to-event outcome, a Kaplan-Meier plot is recommended. | Figure 3 |
| 16. For key multivariable analyses, report estimated effects (eg, hazard ratio) with confidence intervals for the marker and, at least for the final model, all other variables in the model. | Figure 4 |
| 17. Among reported results, provide estimated effects with confidence intervals from an analysis in which the marker and standard prognostic variables are included, regardless of their statistical significance. | Figure 4 |
| 18. If done, report results of further investigations, such as checking assumptions, sensitivity analyses, and internal validation. | External validation: Figure 5 |
| **Discussion** | |
| 19. Interpret the results in the context of the prespecified hypotheses and other relevant studies; include a discussion of limitations of the study. | Discussion section |
| 20. Discuss implications for future research and clinical value. | Discussion section |

# Appendix: FIM databases investigators

Philippe AGAPÉ (Institut Cancérologie de l'Ouest Saint-Herblain), Ahmad AL JIJAKLI (CH Argenteuil), Elisabeth ANDRE-KERNEIS (CH Avignon), Fiorenza BARRACO (HCL Lyon Sud), Frédéric BAUDUER (CH Côte Basque Bayonne), Yohan BENARD (CH Côte Basque Bayonne), Marc BERNARD (CHU Rennes), Charles BESCOND (CH Cholet), Audrey BIDET (CHU Bordeaux), Odile BLANCHET (CHU Angers), Claire BORIES (CH Lens), Françoise BOYER-PERRARD (CHU Angers), Claire CALMETTES (CH Périgueux), Nathalie CAMBIER (CHU Lille), Benjamin CARPENTIER (GHICL Lille), Bruno CASSINAT (APHP Saint-Louis), Emilie CAYSSIALS (CHU Poitiers), Julie CHEVALIER (CH Bretagne Atlantique Vannes), Pascale CONY-MAKHOUL (CH Annecy Genevois), Selim CORM (CH Métropole Savoie Chambéry), Stéphane COURBY (CHU Grenoble), Luc DARNIGE (APHP Hôpital Européen Georges-Pompidou), Benoît DE RENZIS (CHU Clermont-Ferrand), Véronique DE MAS (CHU Toulouse), Jacques DELAUNAY (Hôpital Privé du Confluent Nantes), Guillaume DENIS (CH Rochefort), Yohan DESBROSSES (CHU Besançon), Viviane DUBRUILLE (CHU Nantes), Sophie DUPIRE (CH Bourg-en-Bresse), Gabriel ETIENNE (Institut Bergonié Bordeaux), Jean-Baptiste FOULQUIER (CH Pays de Morlaix), Nathalie GACHARD (CHU Limoges), Cécile GIRARD (CHU Nantes), Stéphane GIRAULT (CHU Limoges), François GIRODON (CHU Dijon), Isabelle GRULOIS (CH Saint-Malo), Philippe GUEUDET (CH Perpignan), Violaine HAVELANGE (Cliniques universitaires Saint-Luc Bruxelles), Sandrine HAYETTE (HCL Lyon Sud), Olivier HERAULT (CHU Tours), Jean-Christophe IANOTTO (CHU Brest), Chloé JAMES (CHU Bordeaux), Jean-Michel KARSENTI (CHU Nice), Jean-Jacques KILADJIAN (APHP Saint-Louis), Florence LACHENAL (CH Pierre-Oudot Bourgoin-Jailleu), Sébastien LACHOT (CHU Tours), Diane LARA (CH Libourne), Kamel LARIBI (CH Le Mans), Marine LE BOUAR (CH Libourne), Yannick LE BRIS (CHU Nantes), Lenaïg LE CLECH (CH Cournouaille Quimper), Jérémie LECARDEUX (CH Périgueux), Isabelle LEDUC (CH Abbeville), Laurence LEGROS (APHP Bicêtre), Cécile LEYRONNAS (GHM Grenoble), François LIFERMANN (CH Dax Côte-d'Argent), Eric LIPPERT (CHU Brest), Marie LOOSVELD (APHM La Timone), Damien LUQUE PAZ (CHU Angers), Antoine MACHET (CHU Tours), Sandra MALAK (Institut Curie Saint-Cloud), Olivier MANSIER (CHU Bordeaux), Marc MAYNADIÉ (CHU Dijon), Clémence MEDIAVILLA (CHU Bordeaux), Fanny MÉNARD (CH Côte Basque Bayonne), Cédric MÉNARD (CHU Rennes), Mélanie MERCIER (CH Bretagne Atlantique Vannes), Mathieu MEUNIER (CHU Grenoble), Isabelle MIGNON (CH Cholet), Pierre-Emmanuel MORANGE (APHM La Timone), Jean-François MOSNIER (CHU Nantes), Pascal MOSSUZ (CHU Grenoble), Marc MULLER (CHRU Nancy), Anne MURATI (Institut Paoli-Calmettes Marseille), Christophe NICOL (CH Pays de Morlaix), Franck-Emmanuel NICOLINI (Centre Léon Bérard Lyon), Mario OJEDA-URIBE (GHR Mulhouse Sud-Alsace), Corentin ORVAIN (CHU Angers), Céline PANGAULT (CHU Rennes), Anne PARRY (CH Annecy Genevois), Amélie PENOT (CHU Limoges), Isabelle PLANTIER (CH Roubaix), Anna RAIMBAULT (CHU Poitiers), Dana RANTA (CHRU Nancy), Vincent REBIÈRE (CH Pays de Morlaix), Matthieu RESCHE-RIGON (APHP Saint-Louis), Jérôme REY (Institut Paoli-Calmettes Marseille), Jean-Baptiste ROBIN (CH Côte Basque Bayonne), Philippe RODON (CH Périgueux), Johann ROSE (CH Le Mans), Lydia ROY (APHP Henri-Mondor), Arnaud SAINT-LÉZER (CH Mont-de-Marsan), Laurence SANHES (CH Perpignan), Marc SIMON (CH Valenciennes), Borhane SLAMA (CH Avignon), Ivan SLOMA (APHP Henri-Mondor), Juliette SORET-DULPHY (APHP Saint-Louis), Léa SUREAU (CHU Angers), Suzanne TAVITIAN (CHU Toulouse), Valérie UGO (CHU Angers), Fabienne VACHERET (CH Perpignan), Anne VEKHOFF (APHP Saint-Antoine), Geoffroy VENTON (APHM la Timone), Céline VERSTUYFT (APHP Bicêtre), Jean-François VIALLARD (CHU Bordeaux), Bruno VILLEMAGNE (CHD Vendée), Anouk WALTER-PETRICH (APHP Saint-Louis), Mathieu WEMEAU (CH Arras), Lise WILLEMS (APHP Cochin).
